# B4GALT1 expression predicts prognosis and adjuvant chemotherapy benefits in muscle-invasive bladder cancer patients

**DOI:** 10.1186/s12885-018-4497-0

**Published:** 2018-05-24

**Authors:** Huyang Xie, Yu Zhu, Junyu Zhang, Zheng Liu, Hangcheng Fu, Yifan Cao, Gaoxiang Li, Yijun Shen, Bo Dai, Jiejie Xu, Dingwei Ye

**Affiliations:** 1 Department of Urology, Fudan University Shanghai Cancer Center, Shanghai Medical College, Fudan University, 270 Dong’an Road, Shanghai, 200032 China; 2 Department of Oncology, Shanghai Medical College, Fudan University, Shanghai, China; 3 Department of Biochemistry and Molecular Biology, School of Basic Medical Sciences, Fudan University, 138 Yixuyuan Road, Shanghai, 200032 China

**Keywords:** Adjuvant chemotherapy, B4GALT1, Muscle-invasive bladder cancer, Overall survival, Prognosis

## Abstract

**Background:**

The expression alterations of B4GALT1 have been noted in some types of cancer and they are related to cancer cell proliferation, invasiveness, metastasis, and drug resistance. We aimed to establish the expression of B4GALT1 in bladder cancer and its connection to patient outcomes, as well as forecasting the advantages of adjuvant chemotherapy (ACT) in patients with muscle-invasive bladder cancer (MIBC).

**Methods:**

There were 142 and 112 MIBC patients who were consecutively recruited and treated via radical cystectomy from 2008 to 2012 in Shanghai Zhongshan Hospital and Fudan University Shanghai Cancer Center (FUSCC), respectively. Tissue microarrays (TMAs) were constructed in triplicate from specimens that had been fixed in formalin and embedded in paraffin samples. Immunohistochemistry was conducted to evaluate B4GALT1 expression in tumor cores, the connection between B4GALT1 expression and patients’ clinical characteristics, and clinical results.

**Results:**

B4GALT1 expression was not connected to clinical prognosis markers, but it was linked to overall survival (OS) (*P* = 0.013 and *P* = 0.010, respectively) in the two groups. Moreover, the high levels of B4GALT1 expression were independent indicators of poor OS (*P* = 0.026 and *P* = 0.046, respectively). Inclusion of B4GALT1 in the prognostic model revealed a greater predictive accuracy than the primary models. In addition, no differences were observed between B4GALT1 expression (low vs. high) and CD8+ T cell infiltration density (number/cm^2^) within tumor cores, but there was a positive Pearson correlation between B4GALT1 expression and expression of inhibitory receptor ligands, such as PD-L1 and CTLA4. Most significantly, the advantage of ACT noted in pT3/4 or N+ bladder cancer patients with low B4GALT1 expression was greater than in patients with a high B4GALT1 expression.

**Conclusions:**

Our evaluation indicated that B4GALT1 may be a possible prognosticator of MIBC, and it may be a predictive marker for the choice of ACT in pT3/4 or N+ patients.

**Electronic supplementary material:**

The online version of this article (10.1186/s12885-018-4497-0) contains supplementary material, which is available to authorized users.

## Background

The fourth most frequently diagnosed cancer, bladder cancer has an occurrence rate of about 7%, and is the eighth most frequent cause of mortality in men (about 4%) [1]. In 2015, it was predicted that there would be 80,500 new bladder cancer cases, with an estimate for deaths of both sexes in China of 32,900 [2]. Even though substantial advancements have been made in bladder cancer treatments, patient prognosis remains poor due to the heterogeneity of the features of this disease [3]. While radical cystectomy is curative in patients with muscle-invasive bladder cancer (MIBC), about 50% of patients experience metastatic recurrence and eventually die from the disease [4]. Each of the urological guidelines suggests neoadjuvant chemotherapy for MIBC patients; however, some evaluations have shown that just 1–15% of MIBC patients are administered neoadjuvant chemotherapy [5]. These same evaluations show that adjuvant chemotherapy (ACT) is given as frequently—or more frequently—than neoadjuvant chemotherapy in daily clinical practice. This outcome has led to the requirement of a precise prognosis evaluation following radical surgery, which is necessary for decision-making about treatment, patient counseling, and assistance in defining ACT indications [6].

Glycans take part in various aspects of the immune response and have been reported to be involved in cancer immune surveillance [7]. Altered cells can be removed by immune effector cells, causing immune selection of tumor cell variants with lowered immunogenicity and resistance to immune effector cells, such as CD8+ T cells and NK cells [8]. Not long ago, an evaluation demonstrated that the ratio of CD8 to Treg tumor-infiltrating lymphocytes is related to the reaction to cisplatin-based neoadjuvant chemotherapy in MIBC patients, which indicates that the immune system has a pivotal part in platinum-based chemotherapy’s effectiveness in treating bladder cancer [9].

The β4-galactosyltransferase (B4GALT) family owns seven members and each of those members possesses distinct biological functions on account of different acceptor specificity, tissue distribution, chronological expression [10]. B4GALT1 conjugates galactose to the outer arm of N-acetylglucosamine in N-linked oligosaccharides of IgG, and B4GALT1 levels are connected to a continuation of the terminal galactose in IgG [11], relating it to the adaptive immune reaction. The significance of glycan β4-galactosylation of the glycans is well known, and this galactosylation could be part of numerous biological activities, such as cancer advancement. The expression alteration of the B4GALTs have been documented in some types of cancer, such as breast cancer [12], colon cancer [11], hepatocellular cancer [13], lung cancer [14], leukemia [15], neuroblastoma [16], and prostate cancer [17] and are connected to cancer cell proliferation, invasiveness, metastasis, and drug resistance.

As our prior evaluations on B4GALT1 in leukemia [15], hepatocellular cancer [13], and renal cancer [18] indicated its pivotal part in cancer biology, we used IHC to analyze B4GALT1 expression in MIBC clinical samples and to determine its connection to clinicopathological features and patients’ overall survival (OS). We further used subgroup analysis, by combining the two cohorts of pT3/4 or N+ patients, to evaluate the association of this biomarker for the benefit from adjuvant chemotherapy.

## Methods

### Patients and clinical databases

Two consecutive groups of 142 and 112 bladder cancer patients that had been treated via radical cystectomy between March 2008 and December 2012 in Shanghai Zhongshan Hospital and Fudan University Shanghai Cancer Center were chosen retrospectively. The clinical characteristics of patients, their laboratory data, and treatment strategies were acquired from in-patient medical records, and follow-up information was gathered by trained nurses. The evaluation was authorized by the Clinical Research Ethics Committee of Fudan University Shanghai Cancer Center and Zhongshan Hospital, Fudan University. All of the participants in this evaluation were well informed about the details, and informed consent was acquired. Participants were chosen based on the criteria: 1) patients were at least 18 years of age; 2) there was a verified histopathological diagnosis; and 3) TNM classification was re-examined based on the 2010 AJCC. Those patients received neoadjuvant chemotherapy (eight and 11 in the two cohorts, respectively) were excluded from analysis. Additional inclusion criteria were the following: tissue blocks embedded in paraffin could be used for immunohistochemical staining and full results data were accessible. OS was determined from the date of surgery to the date of death or last follow-up visit. An experienced radiologist evaluated responses and advancement, which were established by typical criteria. The course of treatment about ACT and after recurrence was platinum based chemotherapy followed the CUA guideline. Both cohorts last follow up ended on July 2016, and the median follow-up for the two cohorts was 34.0 and 64.5 months, respectively.

TCGA dataset and RNAseq expression data from Bladder Urothelial Carcinoma (BLCA) samples were acquired from the TCGA data portal (https://portal.gdc.cancer.gov/). Tumor transcriptomic profiles of 20,534 genes were measured in 436 primary bladder cancer patients, but only 407 MIBC patients with intact clinical information, especially the follow-up data, were included in this study. The CIBERSORT method was applied to the TCGA database to analyze the difference between B4GALT1 and tumor CD8+ T cell infiltration density, which could infer leukocyte representation in bulk tumor transcriptomes [19].

### Immunohistochemistry

The construction of tissue microarrays was carried out as previously detailed [20]. Immunohistochemical staining was carried out with primary anti-B4GALT1 antibody (diluted 1:100; HPA010806; Sigma-Aldrich). Sample staining intensity was assessed by two separate pathologists blinded to patient clinicopathological data and clinical results. A semi-quantitative immunoreactivity scoring (IRS) system was utilized for this examination, as documented elsewhere [21], and it varied from 0 to 300, deriving from the multiplication of intensity of immunohistochemical staining (0, no staining; 1, weak; 2, moderate; and 3, strong) and percentage of positive tumor cells (10 points for each 10% increment; the percentage of positive tumor cells ranged from 0 to 100). X-tile software, version 3.6.1 (Yale University, New Haven, Connecticut) was used to choose the optimum cut-off scores of 120 and 136 for the staining intensities to divide patients into high or low B4GALT1 expression cohorts, respectively.

### Statistical analysis

MedCalc 15.8 and Stata 12.0 were utilized for statistical analysis. Categorical data were examined with Fisher exact or chi-square tests. Numerical data were evaluated with the Student’s *t* test. Subgroup OS curves were determined by the Kaplan-Meier technique and contrasted via log-rank test. We utilized univariate and multivariate Cox proportional hazard models to examine the HR and 95% CI. Prognostic factor accuracy was examined by Harrell’s concordance index (C-index). Moreover, the Akaike information criterion (AIC) value was calculated to assess the prognostic models’ discriminatory capabilities, and decreased AIC values indicated greater predictive ability. Each of the statistical tests was two-sided, and *P* < 0.05 was established as being statistically significant.

## Results

### B4GALT1 intensity by IHC and its connection to MIBC pathological characteristics

**Fig. 1:**
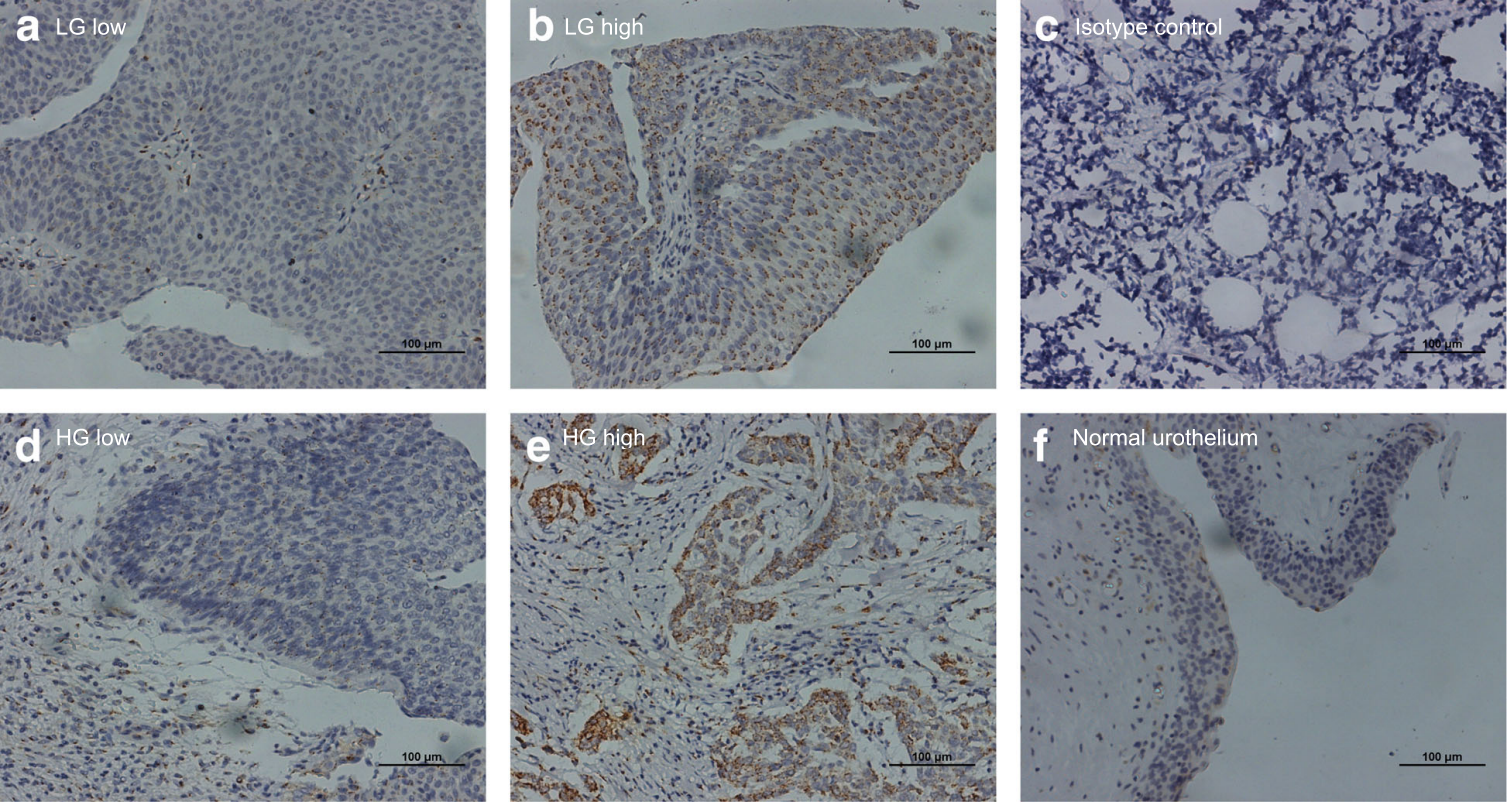
B4GALT1 expression in muscle-invasive bladder cancer (MIBC) tissues. Representative B4GALT1 immunohistochemical images of low expression (**a**) and high expression (**b**) in low pathological grade MIBC tissues; representative B4GALT1 immunohistochemical images of low expression (**d**) and high expression (**e**) in high pathological grade MIBC tissues; negative image by isotype control in bladder tissue (**c**); representative B4GALT1 immunohistochemical image in normal urothelium (**f**). Scale bars (black lines) = 100 um. (Magnification 200×)

To evaluate if B4GALT1 expression is connected to MIBC evolution and advancement, we initially examined its expression by IHC in the training and validation groups. As shown in Fig. 1b and e, B4GALT1-positive staining was mainly found in the cytoplasm and shown as a dot-shaped stain. We also presented the negative control in Fig. 1c, and normal urothelium showed a very low even negative expression of B4GALT1 in Fig. 1f. Urothelium tumor grade did not associate with the expression of B4GALT1, as presented in Fig. 1a and b both were low grade tumor with low and high expression respectively, while high grade tumor in Fig. 1d and e as the same. Based on the cut-off value obtained from the IRS score by X-tile discussed in the Methods section, we divided the two groups into low (*n* = 55 and *n* = 57) and high (*n* = 87 and *n* = 55) B4GALT1 expression cohorts, respectively.

In-depth patient attributes and the relationship between B4GALT1 expression and patient clinicopathological characteristics are included in Table 1. Seventy-five patients (52.8%) died in the training group due to all causes throughout the follow-up period, while 48 patients (42.9%) in the validation group died. The two groups were largely paired for the primary pathological characteristics as revealed in the Additional file 1: Table S1. B4GALT1 expression was significantly connected to tumor grade in the training group but not in the validation group (*P* = 0.017 and *P* = 0.950, respectively; Table 1). This may explain by few patients with low grade tumor in both cohorts (24 and 11 in the two cohorts, respectively), as most patients treated by radical cystectomy were high risk superficial bladder tumor or MIBC. In addition, the expression of B4GALT1 was not associated with patients’ age or gender and not with other confirmed prognosis factors, such as tumor size, pT stage, lymphovascular invasion (LVI) or Charlson Comorbidity Index (CCI) in both the training cohort and validation cohort (all *P* > 0.05 showed in Table 1).

**Table 1:** Association between B4GALT1 expression and patient characteristics

Characteristic	Training set (*n* = 142)					Validation set (*n* = 112)				
	Patients		B4GALT1 expression			Patients		B4GALT1 expression		
	NO.	%	Low	High	*P*	NO.	%	Low	High	*P*
Age, years					0.614					0.763
Mean ± SD	62.4 ± 10.8		61.8 ± 10.9	62.7 ± 10.8		61.5 ± 8.7		61.8 ± 9.7	61.3 ± 7.6	
Range, median	30–82,62		34–82,62	30–82,62		35–81,62		35–81,62	46–75,61	
Gender					0.925					0.581
Male	118	83.1	45	73		97	86.6	48	49	
Female	24	16.9	10	14		15	13.4	9	6	
Tumor size, cm					0.053					0.411
Mean ± SD	3.8 ± 1.9		4.2 ± 2.1	3.6 ± 1.7		3.8 ± 1.8		3.9 ± 2.0	3.7 ± 1.6	
Range	0.5–10.0		1.0–10.0	0.5–8.0		0.8–9.5		0.8–9.5	1.3–9.0	
pT stage					0.971					0.720
T2	68	47.9	27	41		49	43.8	27	22	
T3	48	33.8	18	30		49	43.8	23	26	
T4	26	18.3	10	16		14	12.4	7	7	
Grade					0.017					0.950
low	24	16.9	15	9		11	9.8	6	5	
high	118	83.1	40	78		101	90.2	51	50	
LVI					0.157					0.741
absent	53	37.3	25	28		78	69.6	41	37	
present	89	62.7	30	59		34	30.4	16	18	
CCI					0.116					0.263
≤ 1	47	33.1	23	24		44	39.3	19	25	
≥ 2	95	66.9	32	63		68	60.7	38	30	

### High B4GALT1 expression is connected to worse OS in MIBC patients, and B4GALT1 expression is a separate indicator of poor survival

**Fig. 2:**
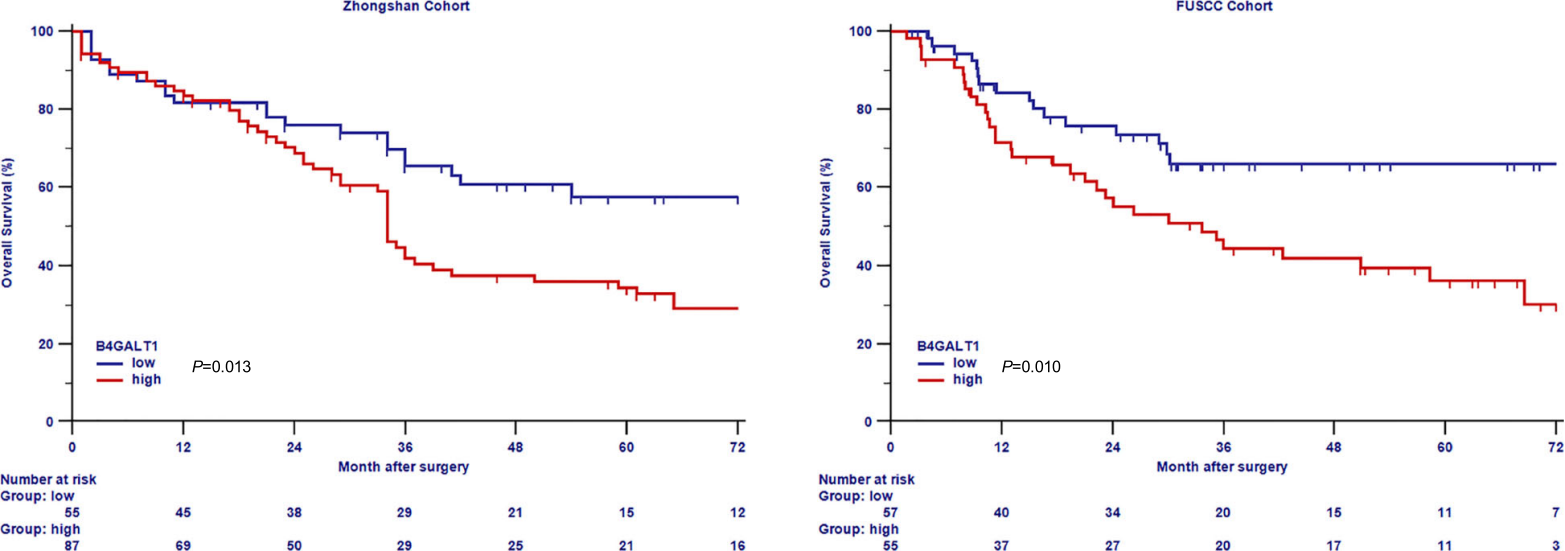
Overall survival (OS) analysis of patients with MIBC based on B4GALT1 expression. Kaplan-Meier analysis of OS in training cohort (*n* = 142) (left) and in validation cohort (*n* = 112) (right). *P* value was calculated by log-rank test

Kaplan–Meier survival analysis was used to contrast OS based on B4GALT1 expression. Patients who exhibited high levels of B4GALT1 expression had a significantly poorer OS in the training and validation groups (log-rank test *P* = 0.013 and *P* = 0.010, respectively; Fig. 2a and b). To examine if this discovery was separate from well-established prognostic indicators, such as pathological T stage, tumor grade, LVI, and CCI, we conducted Cox proportional hazard analyses (univariate and multivariate) of each of the clinicopathological variables with B4GALT1 expression.

Univariate Cox regression analysis showed that tumor pT stage (pT4 vs. pT2, HR = 1.765, 95%CI: 1.005~ 3.100, *P* = 0.049) and B4GALT1 expression (HR = 1.849, 95%CI: 1.126~ 3.035, *P* = 0.016) were associated with OS in the training cohort (Table 2). However, in the validation cohort, patients’ age (HR = 1.062, 95%CI: 1.020~ 1.105, *P* = 0.004), pT stage (pT3 vs. pT2, HR = 2.708, 95%CI:1.426~ 5.142, *P* = 0.003); pN stage (HR = 1.626, 95%CI: 1.194~ 2.213, *P* = 0.002); LVI (HR = 1.926, 95%CI: 1.073~ 3.456, *P* = 0.029); TNM stage (III vs. II, HR = 3.291, 95%CI: 1.471~ 7.362, *P* = 0.004; IV vs. II, HR = 6.290, 95%CI: 2.874~ 13.768, *P* < 0.001), and B4GALT1 (HR = 2.158, 95%CI: 1.187~ 3.923, *P* = 0.012) were identified as risk factors.

**Table 2:** Univariate and multivariate Cox regression analysis of overall survival of the two cohorts

	Univariate			Multivariate		
	HR	95%CI	*P*	HR	95%CI	*P*
Training cohort						
Age(continuous)	1.006	0.986 ~ 1.025	0.574	1.004	0.985 ~ 1.024	0.661
Gender(male vs female)	0.697	0.383 ~ 1.268	0.240			
B4GALT1(high vs low)	1.849	1.126 ~ 3.035	0.016	1.773	1.075 ~ 2.924	0.026
CCI(≥2 vs ≤1)	1.154	0.712 ~ 1.872	0.563			
LVI(present vs absent)	1.449	0.898 ~ 2.339	0.131			
pN(N+ vs N-)	1.651	0.718 ~ 3.795	0.240			
pT stage						
pT3 vs pT2	1.063	0.625 ~ 1.808	0.824			
pT4 vs pT2	1.765	1.005 ~ 3.100	0.049			
TNM stage						
III vs II	1.278	0.797 ~ 2.051	0.312	1.220	0.759 ~ 1.961	0.414
IV vs II	1.877	0.784 ~ 4.492	0.159	1.636	0.680 ~ 3.938	0.274
Validation cohort						
Age(continuous)	1.062	1.020 ~ 1.105	0.004	1.050	1.006 ~ 1.095	0.025
Gender(male vs female)	0.929	0.369 ~ 2.337	0.876			
B4GALT1(high vs low)	2.158	1.187 ~ 3.923	0.012	1.862	1.016 ~ 3.413	0.046
CCI(≥2 vs ≤1)	1.847	0.993 ~ 3.438	0.054			
LVI(present vs absent)	1.926	1.073 ~ 3.456	0.029	1.060	0.458 ~ 2.452	0.893
pN(N+ vs N-)	1.626	1.194 ~ 2.213	0.002			
pT stage						
pT3 vs pT2	2.708	1.426 ~ 5.142	0.003			
pT4 vs pT2	2.517	0.967 ~ 6.550	0.060			
TNM stage						
III vs II	3.291	1.471 ~ 7.362	0.004	2.555	1.126 ~ 5.796	0.026
IV vs II	6.290	2.874 ~ 13.768	< 0.001	5.230	1.962 ~ 13.943	0.001

After that, multivariate Cox regression analysis, including potential risk factors determined by univariate Cox analysis, was conducted. In addition to TNM stage, age, and LVI, B4GALT1 was shown to be an independent prognostic factor for OS (shown in Table 2, dichotomous B4GALT1: HR = 1.773, 95%CI: 1.075~ 2.924, *P* = 0.026 in training cohort; HR = 1.862, 95%CI: 1.016 ~ 3.413, *P* = 0.046 in validation cohort).

### Prognostic models with B4GALT1 expression: Extension for MIBC patients

To additionally evaluate the prognostic ability of B4GALT1 expression as a prognosticator, we created prognostic models that combine B4GALT1 expression with TNM stage and tumor grade by C-index for comparison of their prognostic reliability. As shown in Table 3, in the training group, the C-indices were 0.644 and 0.700 when evaluated with B4GALT1 alone and TNM + Grade, respectively, and they were enhanced to 0.744 when the dichotomous B4GALT1 expression signature was placed in the combination model. Similarly, in the validation group, the C-index was enhanced from 0.637 and 0.679 to 0.746 when dichotomous B4GALT1 signature was placed into the model. Moreover, *P* values suggesting the statistical significance of C-indices of the amalgamated TNM + Grade + B4GALT1 model vs. only B4GALT1 or TNM + Grade were all < 0.05 in the two cohorts.

**Table 3:** Comparison of the accuracy of the prognostic models and B4GALT1 expression for overall survival

Model		Training cohort			Validation cohort	
	C-index	95% CI	*P* value	C-index	95% CI	*P* value
B4GALT1 (high vs. low)	0.644	0.558 ~ 0.741	0.016	0.637	0.548 ~ 0.726	0.028
TNM + Grade	0.700	0.606 ~ 0.783	0.031	0.679	0.591 ~ 0.766	0.039
TNM + Grade + B4GALT1	0.744	0.653 ~ 0.822		0.746	0.648 ~ 0.835	

Note: *P* values indicating statistical significance of C-indices of combined TNM + Grade + B4GALT1 vs only B4GALT1 or TNM + Grade

### B4GALT1 expression and the benefit from ACT in pT3/4 or N+ patients

**Fig. 3:**
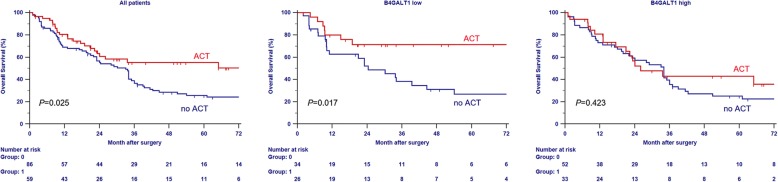
Relationship between B4GALT1 expression and benefit from adjuvant chemotherapy (ACT). Survival curves for pT3/4 or N+ patients with ACT and without ACT in combined cohorts (left) and in low B4GALT1 expression subgroup (middle) and in high B4GALT1 expression subgroup (right). *P* value was calculated by log-rank test

For patients of pT3/4 or N+ bladder cancer, ACT has improved survival rate and has become more often used in clinical practice than neoadjuvant chemotherapy. In our datasets, few patients used neoadjuvant chemotherapy but 41% patients received ACT. To explore the association of the B4GALT1 expression signature with response to platinum-based ACT, subgroup analysis was performed in these patients. As presented in Fig. 3, the combined patients with pT3/4 or N+ diseases showed better survival with ACT compared to without ACT (log-rank test *P* = 0.025). By incorporating B4GALT1 signature into ACT information, the low B4GALT1 expression subgroup, patients with ACT had more survival benefit than patients without ACT (log-rank test *P* = 0.017). On the contrary, high B4GALT1 group patients did not have benefit from ACT (log-rank test *P* = 0.423).

### B4GALT1 expression in MIBC was associated with tumor immunosuppressive status

**Fig. 4:**
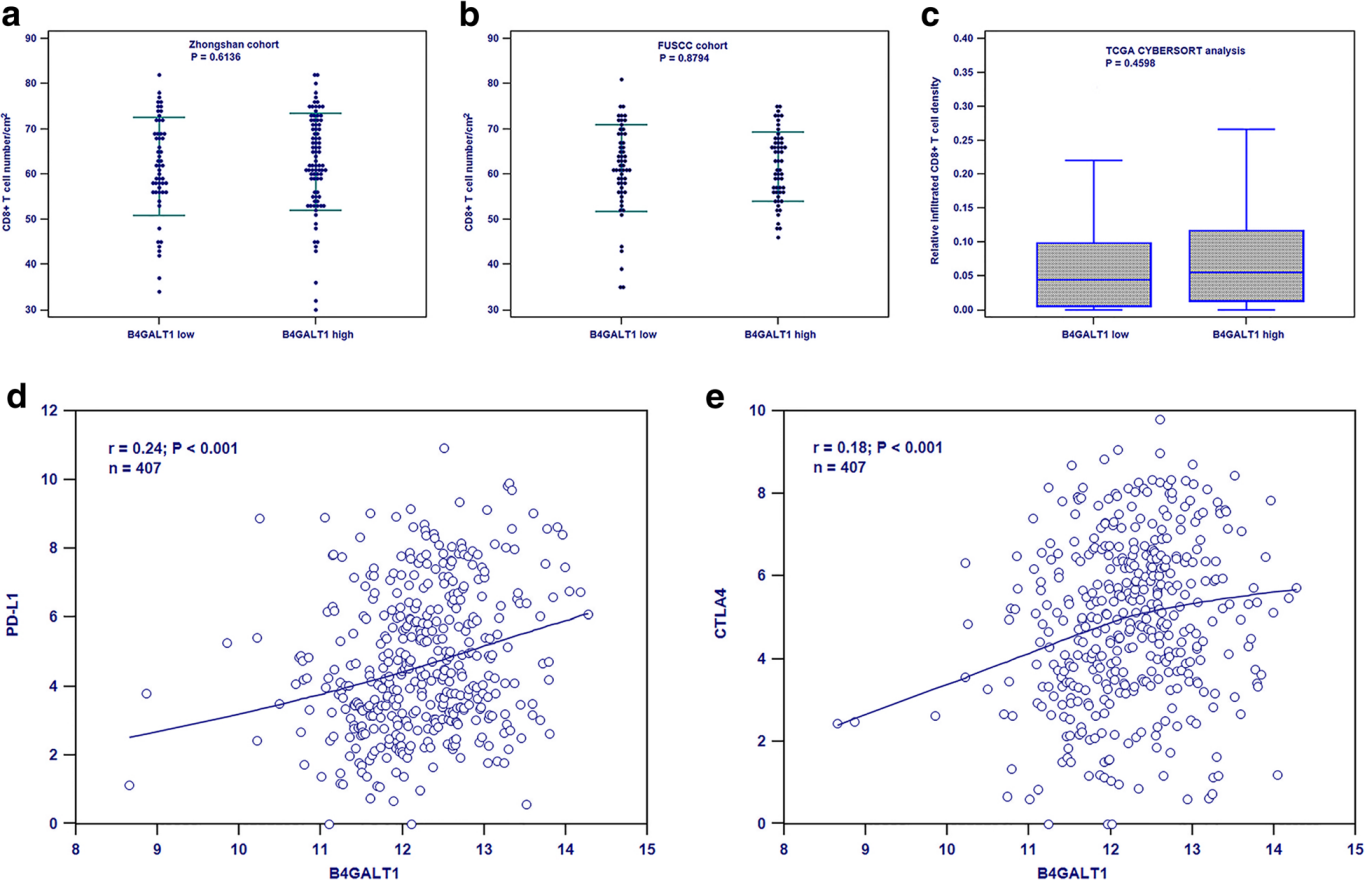
B4GALT1 expression in MIBC was associated with tumor immunosuppressive status. No differences between B4GALT1 expression (low vs. high) and CD8+ T cell infiltration density (number/cm^2^) within tumor cores in Zhongshan cohort (**a**), FUSCC cohort (**b**) and TCGA_BLCA cohort calculated by CYBERSORT method (**c**); the positive Pearson correlation between B4GALT1 and PD-L1 expression (**d**) and CTLA4 expression (**e**) in TCGA dataset

To examine the mechanism of low B4GALT1 expression that provides patients with advantages from ACT, we considered that the immune system, in particular the efficient immune cells, has a crucial part in the reaction of bladder cancer to chemotherapy. Unfortunately, no differences between B4GALT1 expression (low vs. high) and CD8+ T cell infiltration density (number/cm^2^) within tumor cores in the Zhongshan cohort (Fig. 4a), FUSCC cohort (Fig. 4b) and TCGA_BLCA cohort calculated by CYBERSORT method (Fig. 4c). Since glycans control different features of the immune reaction hindering tumor surveillance, we determined if the B4GALT1 expression was linked to inhibitory receptor ligands in tumors, such as PD-L1 and CTLA4, whose humanized antibodies have demonstrated groundbreaking clinical outcomes in patients with bladder cancer. And positive Pearson correlation between B4GALT1 and PD-L1 expression in Fig. 4d (*r* = 0.24, *P* < 0.001) and CTLA4 expression in Fig. 4e (*r* = 0.18, *P* < 0.001) were found in the TCGA dataset.

## Discussion

Of the constructed bladder cancer prognostic models, the American Joint Committee on Cancer (AJCC) TNM staging system, which has been properly confirmed, is the most broadly utilized prognostic model to forecast results in patients treated with radical cystectomy. Nevertheless, a large weakness of this system is the difficulty in integrating new clinical information, including molecular markers or more elaborate bioinformatics. Moreover, the established staging systems are less reliable than several of the prediction models integrating clinical data in the time of personalized medicine [22]. In our evaluation, the integration of B4GALT1 into TNM staging might elevate the risk stratification of MIBC patients. We found a negative correlation between B4GALT1 and MIBC outcome and validated B4GALT1 as an independent prognosis marker in two independent cohorts for OS in MIBC patients.

Clinical practice guidelines provide diverse guidance to ACT in MIBC, with the National Comprehensive Cancer Network guidelines encouraging ACT as a low (2B) suggestion [23], but the European Association of Urology guidelines indicate that there is not adequate data to encourage the typical administration of chemotherapy [24]. In a big observational evaluation, ACT was connected to increased survival in patients with locally progressed bladder cancer postcystectomy [25]. Some studies reveal that ACT is administered more often than neoadjuvant chemotherapy in real clinical practice [5]. This situation is consistent with our study that 41% locally advanced patients received ACT but few patients to neoadjuvant chemotherapy. So the selection of proper patients for ACT has its practical significance. We found that among locally advanced patients with ACT or without ACT, the former group had survival benefit. When incorporating B4GALT1 signature, low B4GALT1 expression subgroup patients with ACT had more survival benefit than patients without ACT, but high B4GALT1 expression subgroup patients had no benefit from ACT, indicating that B4GALT1 expression could be an important factor for the efficiency of chemotherapy. This will assist in choosing and handling patients who will be administered ACT.

To explore the underlying mechanisms of low B4GALT1 expression patients benefit from ACT, we figured that the efficient immune cells have a pivotal part in the reaction of bladder cancer to chemotherapy, which is supported by a recent study [9]. But we found no difference of CD8+ T cell infiltration density in two B4GALT1 patients group in our cohorts and neither in TCGA dataset. As glycans regulate immune response interfering with tumor surveillance [26], we explored whether the B4GALT1 expression was associated with PD-L1 and CTLA4, whose humanized antibodies have shown unprecedented clinical results in patients with MIBC, and both had positive spearman correlation, indicating that tumor cell of high expression B4GALT1 could form more potent immunosuppressive microenvironment. Engagement of inhibitory receptors (IRs) on interaction with their cognate ligands leads to dimming of T-cell receptor signaling, resulting in a reduction in immune responses to antigens [27]. IRs expression has been associated with T-cell exhaustion in cancers, leading to T-cell impotence in tumor eradication [28]. Low doses chemotherapy agents, including cisplatin, selectively inhibit regulatory and suppressor cells [29]. To sum up, these results have given us the rationale for combining checkpoint receptor inhibition with chemotherapy in the future.

The biggest limitations of this evaluation are its retrospective design and relatively minute sample size. In addition, each of the specimens was gathered from two tertiary referral hospitals, so the outcomes should be further confirmed or reassessed in bigger datasets and external heterogeneous groups. Additional expanded population evaluation on this and prospective external validation are needed.

## Conclusions

Our evaluation indicated that B4GALT1 may be a possible prognosticator of MIBC, and it may be a marker for predicting choice of adjuvant chemotherapy in locally progressed patients. High expression of B4GALT1 could bring about more potent immunosuppressive microenvironment, and could help make better selection of MIBC patients for combined therapy with chemotherapy and immunotherapy.

## Additional file

Additional file 1: **Table S1.** Training and validation sets patients’ characteristics. (DOCX 17 kb)

## Abbreviations

ACT: Adjuvant chemotherapy
B4GALT1: Beta-1,4-galactosyltransferase
CCI: Charlson Comorbidity Index
FUSCC: Fudan University Shanghai Cancer Center
IRs: Inhibitory receptors
LVI: Lymphovascular invasion
MIBC: Muscle-invasive bladder cancer
OS: Overall survival
TMAs: Tissue microarrays
