# Supplementary material for: B4GALT1 expression predicts prognosis and adjuvant chemotherapy benefits in muscle-invasive bladder cancer patients
Source: BMC Cancer. 2018 May 24;18:590. doi: 10.1186/s12885-018-4497-0 (PMC5968541; doi:10.1186/s12885-018-4497-0)
Supplement: Supplementary file 1 — Table S1. Training and validation sets patients’ characteristics. (DOCX 17 kb) [file 12885_2018_4497_MOESM1_ESM.docx]

Table S1. Training and validation sets patients’ characteristics.

|  | **Training set Validation set *P* value** | | | | | | | |
| --- | --- | --- | --- | --- | --- | --- | --- | --- |
| Age, years |  |  |  |  | 0.496 |  |  |  |
| Mean±SD | 62.4±10.8 |  | 61.5±8.7 |  |  |  |  |  |
| Gender |  |  |  |  | 0.552 |  |  |  |
| Male | 118 |  | 97 |  |  |  |  |  |
| Female | 24 |  | 15 |  |  |  |  |  |
| Tumor size, cm |  |  |  |  | 0.999 |  |  |  |
| Mean±SD | 3.8±1.9 |  | 3.8±1.8 |  |  |  |  |  |
| Range | 0.5-10.0 |  | 0.8-9.5 |  |  |  |  |  |
| pT stage |  |  |  |  | 0.202 |  |  |  |
| T2 | 68 |  | 49 |  |  |  |  |  |
| T3 | 48 |  | 49 |  |  |  |  |  |
| T4 | 26 |  | 14 |  |  |  |  |  |
| Grade |  |  |  |  | 0.149 |  |  |  |
| low | 24 |  | 11 |  |  |  |  |  |
| high | 118 |  | 101 |  |  |  |  |  |
| LVI |  |  |  |  | <0.001 |  |  |  |
| absent | 53 |  | 78 |  |  |  |  |  |
| present | 89 |  | 34 |  |  |  |  |  |
| CCI |  |  |  |  | 0.374 |  |  |  |
| ≤1 | 47 |  | 44 |  |  |  |  |  |
| ≥2 | 95 |  | 68 |  |  |  |  |  |
